# Toll-like receptor 9 (*TLR9*) genetic variants rs187084 and rs352140 confer protection from Behcet’s disease among Iranians

**DOI:** 10.1186/s41927-024-00382-x

**Published:** 2024-03-14

**Authors:** Zahra Tadayon, Seyed Abolhassan Shahzadeh Fazeli, Nasser Gholijani, Gholamreza Daryabor

**Affiliations:** 1 Department of Molecular and Cellular Biology, Faculty of Basic Sciences and Advanced Technologies in Biology, University of Science and Culture, ACECR, Tehran, Iran; 2 Department of Genetics, Reproductive Biomedicine Research Center, Royan Institute for Reproductive Biomedicine, ACECR, Tehran, Iran; 3 Autoimmune Disease Research Center, School of Medicine, Shiraz University of Medical Sciences, PO Box: 71345-1583, Shiraz, Iran

**Keywords:** Autoimmunity, Behçet’s disease, Toll-like receptor 9 (*TLR9*), Single nucleotide polymorphism (SNP)

## Abstract

**Background:**

Behcet’s disease (BD) is a multisystem and multifactorial autoimmune disease characterized by relapsing episodes of oral aphthae, genital ulcers, and ocular and skin lesions. Toll-like receptor 9 (*TLR9*) has pro-inflammatory roles and its genetic variants might be involved in the pathogenesis of inflammatory diseases.

**Methods:**

Two hundred five BD patients and 207 age and sex-matched healthy controls were evaluated for *TLR9* single nucleotide polymorphisms − 1486 T/C (rs187084) and + 2848:G/A (rs352140) using polymerase chain reaction-restriction fragment length polymorphism (RFLP-PCR).

**Results:**

Healthy individuals had a significantly higher frequency of rs187084 AG and AG + GG genotypes than BD patients (*p* = 0.02 and *p* = 0.018; respectively). Of interest, healthy males had a significantly higher frequency of rs187084 AG + GG genotype and G allele than male BD patients (*p* = 0.035 and *p* = 0.045; respectively). However, rs187084 AG genotype and G allele frequencies were significantly higher in male patients with genital aphthous (*p* = 0.01 and *p* = 0.046; respectively). Furthermore, a significantly higher frequency of rs352140 CT and TT + CT genotypes was detected in healthy individuals than in BD patients (*p* = 0.01, and *p* = 0.032; respectively). Such results were also seen in healthy females than female patients (*p* = 0.001, and *p* = 0.004; respectively). Haplotype analysis revealed a significantly higher frequency of A-C and G-C haplotypes among patients and healthy subjects, respectively (*p* = 0.002 and *p* = 0.000; respectively).

**Conclusion:**

Our data suggested that rs187084 AG and AG + GG genotypes and rs352140 CT and TT + CT genotypes protect Iranian individuals from BD but rs187084 AG genotype and G allele predispose male BD individuals to genital aphthous. However, additional studies are required to verify these results.

**Supplementary Information:**

The online version contains supplementary material available at 10.1186/s41927-024-00382-x.

## Introduction

Behcet’s disease (BD) is a complex, multisystem inflammatory condition, mainly characterized by mucocutaneous ulcers, and ocular and vascular involvement. Gastrointestinal and central nervous system (CNS) are involved in most severe cases [1]. The prevalence of BD depends on geographical region. Particularly, the outbreak of BD in the ancient Silk Road, which contains Italy, Turkey, Israel, Saudi Arabia, Iran, China, Korea, and Japan, is higher than in other countries [2, 3]. The disease is distributed equally between men and women but globally, males are more frequently affected than women and experience more severe forms of disease [4, 5]. The exact pathogenic reason for BD is not exactly known but some evidence pointed to crucial roles of immunological abnormalities and environmental factors [6, 7]. With recent progress in genetic research, many genes such as human leukocyte antigen (HLA), especially HLA-B51, have been introduced as candidates that increase the susceptibility to BD [8]. Toll-like receptors (TLRs) which mediate innate immune responses are suspected to play vital roles in autoimmune diseases [9]. Increasing evidence has revealed possible associations between gene expression, single nucleotide polymorphisms (SNP), and copy number variation of TLRs and molecules involved in their signaling and the development of BD [8, 10, 11]. *TLR9* gene which is located on chromosome 3p21.3 is one of the susceptible regions associated with BD [12]. *TLR9* which recognizes cytosine-phosphate-guanosine (CPG) motifs is expressed by B cells and acts as a sensor for bacterial infection to activate the innate immune cells [13, 14]. Besides, autoantibody production and induction of inflammatory cells are modulated through the *TLR9* signaling pathway [14–16]. Previous studies have revealed the importance of *TLR9* gene variants in the pathogenesis of autoimmune diseases such as Systemic lupus erythematosus (SLE) [17–19], rheumatoid arthritis (RA) [20], ankylosing spondylitis (AS) [21], acute myeloid leukemia (AML) [22], Hashimoto’s autoimmune thyroiditis [23], and Graves’ disease (GD) [24]. However, their possible association with BD has provided controversial results [24]. *TLR9* rs187084 and rs352140 are located at the promoter region and second exon, respectively, where they regulate *TLR9* expression [17, 25]. These SNPs are the most functional and their special genotypes might lead to changes in *TLR9* gene expression. Therefore, in this study, the possible association between these *TLR9* genetic variants and the pathogenesis of BD was analyzed among Iranian individuals.

## Materials and methods

### Participants

This study included 205 BD patients, including 89 males and 116 females with a mean age of 36 ± 0.72 years, who were diagnosed based on the International Criteria for Behcet Disease in Hafez Rheumatology Outpatient Clinic, Shiraz, Iran [26]. Pregnant patients and those with malignancy, cancer, and other autoimmune diseases were excluded from the study. Two hundred seven age- and sex-matched healthy blood donors (100 males and 107 females with a mean age of 37 ± 0.4) who had no history of autoimmune or inflammatory disease participated as the healthy controls. Our study was approved by the Ethics Committee of the Shiraz University of Medical Science (Code: IR.SUMS.REC.1400.695) and was conducted following the ethical principles outlined in the Declaration of Helsinki (https://www.wma.net/policies-post/wma-declaration-of-helsinki-ethical-principles-for-medical-research-involving-human-subjects/). Before participation, all the individuals provided written informed consent. The clinical, laboratory, and demographic parameters of participants are presented in Table 1.

**Table 1:** The main demographic, laboratory findings, and clinical characteristics of subjects

Variables	BD patients (*n* = 205)	Control (*n* = 207)
Age (years)	36 ± 0.72	37 ± 0.4
Male/ Female	89 (43.4%)/ 116 (56.6%)	100 (48.3%)/ 107 (51.7%)
Age of onset (year)	30.8 ± 0.8	-
Family history of BD	37 (18.04%)	-
Family history of others	39 (19.02%)	-
Autoimmune disease		-
Smoking	37 (18.04%)	
Positive pathergy test	56 (27.31%)	-
HLA-B27 positive	8 (3.9%)	-
HLA-B51 positive	31 (15.12%)	-
Oral aphthous	177 (86.34%)	-
Genital aphthous	105 (51.12%)	-
Skin manifestations	86 (41.95)	-
Eye manifestations	67 (32.68%)	-
Joint manifestations	111 (54.14%)	-
Neural manifestations	53 (25.85%)	-
Vascular manifestations	13 (6.34%)	-
Cardiac involvement	11 (5.36%)	-
Lung involvement	6 (2.92%)	-
Renal involvement	18 (8.78%)	-
Gastrointestinal involvement	17 (8.29%)	-
WBC (Cells/mm3, Mean ± SD)	7693.8 ± 597.13	-
CRP positive (>6 mg/L)	27 (13.17%)	
ESR (mm, Mean ± SD)	15.3 ± 1.2	-

Data is represented as a Number (percentage)

*BD* Behçet’s Disease, *WBC* White Blood Cells, *CRP* C-Reactive Proteins, *ESR* Erythrocyte Sedimentation Rate

### DNA extraction and SNPs genotyping

A total of 5 mL of whole blood samples in EDTA was collected. Genomic DNA was extracted using the salting out method and was stored at -20˚C until analysis [27]. A polymerase chain reaction followed by restriction fragment length polymorphism (PCR-RFLP) was used for genotyping *TLR9* SNPs (rs187084 and rs352140) [28]. The PCR was done in 10 µl reaction volume containing 2 µl of DNA, 0.7 µM of each primer (Pioneer, South-Korea, Table 2), 0.1 µl Taq DNA polymerase (CinnaGen, Iran), 0.3 µl dNTPs (CinnaGen, Iran), 1 µl of 10X buffer, 0.7 µl MgCl2 and 4.5 µl distilled water. For the detection of DNA contamination, no template control (NTC) was included. The amplification conditions were initial denaturation at 95˚C for 5 min, then 30 cycles of denaturation at 95 °C for 30 s, annealing at 62 °C for 30 s, extension at 72 °C for 30 s, followed by a final extension at 72 °C for 5 min. Subsequently, PCR products were digested and incubated at 37˚C for 24 h. After that, fragments were assessed using 3% agarose gel with DNA safe stain (CinnaGen, Iran) and visualized in a UV transilluminator (Upland, CA) (Supplementary Fig. 1). The sequences of forward and reverse primers for each SNP, required restriction enzyme are shown in Table 2.

**Table 2:** The primer sequence, restriction enzymes, and fragment length of SNPs

SNP	Location	Primers (5’-3’)	Ta (◦C)	Enzyme	Genotypes and fragments (bp)
rs187084	5’ flanking region	F: TCATTCAGCCTTCACTCAGAAA R: ACCTCCCACCCCAGATCT	60° C	*AflII*	GG = 299 AA = 148 + 151 AG = 299 + 148 + 151
rs352140	Exon 2	F: GGCTGTGGATGTTGTTGTG R: GCAGCACCCTCAACTTCAC	58° C	*BstUI*	TT = 360 CC = 132 + 228 CT = 360 + 132 + 228

*Ta* Annealing temperature, *SNP* Single nucleotide polymorphism

### Statistical analysis

All statistical analyses were performed using Statistical Package for Social Sciences (SPSS) and Epi-info 7.2.2.6 software packages. Hardy–Weinberg equilibrium was calculated by Arlequin (version 3.1) software to assess the consistency of the genotype distribution. Comparison of the genotype and allele frequencies of *TLR9* polymorphisms between studied groups was done by a two-tailed person’s Chi-square (χ²) test. Statistical significance in all tests was set at *p* values less than 0.05.

## Results

### Demographic and clinical history of BD patients

Clinical manifestation and demographic data of BD patients and controls are shown in Table 1. These data show no significant differences in age and sex between patients and controls (*p* = 0.511 and *p* = 0.214; respectively).

### Genotype and allele frequencies of TLR9 gene polymorphisms

Both rs187084 and rs352140 followed the Hardy–Weinberg equilibrium (*p* = 0.06 and *p* = 0.78; respectively). A significantly higher frequency of rs352140 CT and TT + CT genotypes was observed in the healthy group compared to the BD patients (*p* = 0.01 and *p* = 0.032; respectively; Table 3). Further analysis revealed such results in healthy females compared with the female patients (*p* = 0.001 and *p* = 0.004; respectively). In the case of rs187084, significantly higher frequencies of AG and AG + GG genotypes were indicated in healthy individuals compared with the patients (*p* = 0.02 and *p* = 0.018; respectively). rs187084 GG + AG genotype combination and G allele frequencies were also significantly higher among healthy males compared with the male BD patients (*p* = 0.035 and *p* = 0.045; respectively). As shown in Table 4, analysis of patients according to their disease manifestation revealed only a significant association between rs187084 AG genotype and G allele and genital aphthous in male BD patients (*p* = 0.01 and *p* = 0.046; respectively).

**Table 3:** Genotype and allele frequencies of *TLR9* gene variants in BD and healthy controls

SNP	BD patients N (%)				Healthy controls N (%)			OR (95%CI)			*p*-value		
		Total	Male	Female	Total	Male	Female	Total	Male	Female	Total	Male	Female
rs187084													
Genotypes	AA	80 (39)	35 (39.3)	45 (38.8)	58 (28)	25 (25)	33 (30.8)	1.00 (reference)	1.00 (reference)	1.00 (reference)			
	AG	96 (46.8)	39 (43.8)	57 (49.1)	116 (56)	52 (52)	64 (59.8)	0.6 0.38–0.92	0.53 0.27–1.03	0.65 0.36–1.15	**0.02**	0.063	0.14
	GG	29 (14.1)	15 (16.9)	14 (12.1)	33 (15.9)	23 (23)	10 (9.3)	0.63 0.34–1.16	0.46 0.2–1.06	1.02 0.4–2.5	0.14	0.07	0.95
Alleles	A	256 (62.4)	109 (61.2)	147 (63.4)	232 (56)	102 (51)	130 (60.8)	1.00 (reference)	1.00 (reference)	1.00 (reference)			
	G	154 (37.6)	69 (38.8)	85 (36.6)	182 (44)	98 (49)	84 (39.2)	0.76 0.58–1.01	0.66 0.43–0.99	0.89 0.61–1.31	0.06	**0.045**	0.56
Genotype combination	GG	29 (14.1)	15 (16.9)	14 (12.1)	33 (15.9)	23 (23)	10 (9.3)	1.00 (reference)	1.00 (reference)	1.00 (reference)			
	AA + AG	176 (85.9)	74 (83.1)	102 (87.9)	174 (78)	77 (77)	97 (90.7)	1.15 0.67–1.97	1.47 0.71–3.04	0.75 0.31–1.77	0.61	0.29	0.51
	AA	80 (39)	35 (39.3)	45 (49.1)	58 (28)	25 (25)	33 (30.8)	1.00 (reference)	1.00 (reference)	1.00 (reference)			
	GG + AG	125 (61)	54 (60.7)	71 (50.9)	149 (72)	75 (75)	74 (69.2)	0.6 0.4–0.91	0.51 0.27–0.95	0.7 0.4–1.22	**0.018**	**0.035**	0.21
rs352140													
Genotypes	CC	57 (28.7)	26 (29.2)	31 (26.7)	39 (18.8)	27 (27)	12 (11.2)	1.00 (reference)	1.00 (reference)	1.00 (reference)			
	CT	100 (48.8)	40 (44.9)	60 (51.7)	129 (62.3)	48 (48)	81 (75.7)	0.53 0.33–0.86	0.86 0.4–1.7	0.29 0.14–0.60	**0.01**	0.68	**0.001**
	TT	48 (23.4)	23 (25.8)	25 (21.6)	39 (18.8)	25 (25)	14 (13.1)	0.84 0.46–1.5	0.9 0.44–2.08	0.69 0.27–1.75	0.57	0.9	0.44
Alleles	C	214 (52.2)	92 (51.7)	122 (52.6)	207 (50)	102 (51)	105 (49)	1.00 (reference)	1.00 (reference)	1.00 (reference)			
	T	196 (47.8)	86 (48.3)	110 (47.4)	207 (50)	98 (49)	109 (51)	0.9 0.69–1.19	0.97 0.65–1.4	0.87 0.6–1.26	0.53	0.89	0.46
Genotype combination	CC	57 (27.8)	26 (29.2)	31 (26.7)	39 (18.8)	27 (27)	12 (11.2)	1.00 (reference)	1.00 (reference)	1.00 (reference))			
	TT + CT	148 (72.2)	63 (70.8)	85 (73.3)	168 (81.2)	73 (73)	95 (88.8)	0.6 0.38–0.96	0.9 0.47–1.7	0.35 0.17–0.72	**0.032**	0.73	**0.004**
	TT	48 (23.4)	23 (25.8)	25 (21.6)	39 (18.8)	25 (25)	14 (13.1)	1.00 (reference)	1.00 (reference)	1.00 (reference)			
	CC + CT	157 (76.6)	66 (74.2)	91 (78.4)	168 (81.2)	75 (75)	93 (86.9)	0.76 0.47–1.22	0.96 0.5–1.84	0.55 0.27–1.12	0.26	0.89	0.099

### Haplotype analysis

Table 5 indicates that A-C haplotype frequency is significantly higher in BD patients (*p* = 0.002) while G-C haplotype is more frequent in the control group (*p* = 0.000) (Table 5).

**Table 4:** Association between genotypes and alleles of rs187084 and rs352140 and complications of BD

	Total	Male	Female	Total	Male	Female	Total	Male	Female	Total	Male	Female
*TLR9* rs187084												
	Oral aphthous											
	Positive			Negative			OR (95% CI)			*p*-value		
AA	67 (37.8)	29 (37.2)	38 (38.4)	1 (33.3)	1 (50.0)	0 (0.0)	1.00 (reference)	1.00 (reference)	1.00 (reference)			
AG	83 (46.9)	35 (44.9)	48 (48.5)	2 (66.7)	1 (50.0)	1 (100)	0.6 0.05–6.9	1.2 0.07–20.1	0.4 0.02–10.6	0.7	0.9	0.6
GG	27 (15.3)	14 (17.9)	13 (13.1)	0 (0.0)	0 (0.0)	0 (0.0)	1.2 0.05–30.9	1.5 0.06–38.5	0.3 0.007–18.5	0.9	0.8	0.6
A	217 (61.3)	93 (59.6)	124 (62.6)	4 (66.7)	3 (75.5)	1 (50.0)	1.00 (reference)	1.00 (reference)	1.00 (reference)			
G	137 (38.7)	63 (40.4)	74 (37.4)	2 (33.3)	1 (25.0)	1 (50.0)	1.3 0.2–6.9	2.03 0.2–19.9	0.6 0.4–9.7	0.8	0.6	0.7
	Genital aphthous											
	Positive			Negative			OR (95% CI)			*p*-value		
AA	34 (32.4)	11 (25.0)	23 (37.7)	32 (47.1)	17 (53.1)	15 (41.7)	1.00 (reference)	1.00 (reference)	1.00 (reference)			
AG	55 (52.4)	24 (54.5)	31 (50.8)	27 (39.7)	10 (31.3)	17 (47.2)	1.9 0.9–3.7	3.7 1.3–1.7	1.2 0.5–2.9	0.6	**0.01**	0.7
GG	16 (15.2)	9 (20.5)	7 (11.5)	9 (13.2)	5 (15.6)	4 (11.1)	1.6 0.6–4.3	2.8 0.7–10.5	1.14 0.3–4.6	0.3	0.13	0.8
A	123 (58.6)	46 (52.3)	77 (63.1)	91 (66.9)	44 (68.7)	47 (65.3)	1.00 (reference)	1.00 (reference)	1.00 (reference)			
G	87 (41.4)	42 (47.7)	45 (36.9)	45 (33.1)	20 (31.3)	25 (34.7)	1.4 0.9–2.2	2.0 1.02–3.9	1.1 0.6–2.01	0.1	**0.046**	0.8
	Skin involvement											
	Positive			Negative			OR (95% CI)			*p*-value		
AA	32 (37.2)	16 (34.8)	16 (40.0)	34 (38.6)	13 (41.9)	21 (36.8)	1.00 (reference)	1.00 (reference)	1.00 (reference)			
AG	36 (41.9)	19 (41.3)	17 (42.5)	46 (52.3)	15 (48.4)	31 (54.4)	0.8 0.4–1.6	1.02 0.4–2.8	0.7 0.3–1.7	0.6	0.9	0.5
GG	18 (20.9)	11 (23.9)	7 (17.5)	8 (9.1)	3 (9.7)	5 (8.8)	2.4 0.9–6.3	2.9 0.7–12.9	1.8 0.5–6.9	0.07	0.1	0.4
A	100 (58.1)	51 (55.4)	49 (61.2)	114 (64.8)	41 (66.1)	73 (64.0)	1.00 (reference)	1.00 (reference)	1.00 (reference)			
G	72 (41.9)	41 (44.60	31 (38.8)	62 (35.2)	21 (33.9)	41 (36.0)	1.3 0.9–2.04	1.6 0.8–3.06	1.1 0.6–2.03	0.2	0.18	0.7
	Eye involvement											
	Positive			Negative			OR (95% CI)			*p*-value		
AA	28 (41.8)	13 (41.9)	15 (41.7)	36 (40.0)	15 (42.9)	21 (38.2)	1.00 (reference)	1.00 (reference)	1.00 (reference)			
AG	30 (44.8)	13 (41.9)	17 (47.2)	38 (42.2)	12 (34.3)	26 (47.3)	1.01 0.5–2.01	1.2 0.4–3.7	0.9 0.4–2.2	0.9	0.7	0.8
GG	9 (13.4)	5 (16.1)	4 (11.1)	16 (17.8)	8 (22.9)	8 (14.5)	0.7 0.3–1.9	0.7 0.2–2.8	0.7 0.2–2.7	0.5	0.6	0.6
A	86 (64.2)	39 (62.9)	47 (65.3)	110 (61.1)	42 (60.0)	68 (61.8)	1.00 (reference)	1.00 (reference)	1.00 (reference)			
G	48 (35.8)	23 (37.1)	25 (34.7)	70 ((38.9)	28 (40.0)	42 (38.2)	0.9 0.6–1.4	0.9 0.4–1.8	0.9 0.5–1.6	0.6	0.7	0.6
	joint involvement											
	Positive			Negative			OR (95% CI)			*p*-value		
AA	44 (39.6)	15 (34.1)	29 (43.3)	25 (36.2)	15 (42.9)	10 (29.4)	1.00 (reference)	1.00 (reference)	1.00 (reference)			
AG	51 (45.9)	21 (47.7)	30 (44.8)	33 (47.8)	14 (40.0)	19 (55.9)	0.9 0.4–1.7	1.5 0.6–4.01	0.5 0.2–1.4	0.7	0.4	0.2
GG	16 (14.4)	8 (18.2)	8 (11.9)	11 (15.9)	6 (17.1)	5 (14.7)	0.8 0.3–2.06	1.3 0.4–4.8	0.6 0.1–2.1	0.7	0.6	0.4
A	139 (62.6)	51 (57.9)	88 (65.7)	83 (60.1)	44 (62.8)	39 (57.3)	1.00 (reference)	1.00 (reference)	1.00 (reference)			
G	83 (37.4)	37 (42.1)	46 (34.3)	55 (39.9)	26 (37.2)	29 (42.7)	0.9 0.6–1.4	0.2 0.6–2.3	0.7 0.4–1.3	0.6	0.5	0.2
	Neural involvement											
	Positive			Negative			OR (95% CI)			*p*-value		
AA	21 (39.6)	9 (39.1)	12 (40.0)	46 (38.0)	20 (36.4)	26 (39.4)	1.00 (reference)	1.00 (reference)	1.00 (reference)			
AG	21 (39.6)	9 (39.1)	12 (40.0)	60 (49.6)	27 (49.1)	33 (50.0)	0.8 0.4–1.6	0.7 0.2–2.2	0.8 0.3–2.03	0.5	0.6	0.6
GG	11 (20.8)	5 (21.7)	6 (20.0)	15 (12.4)	8 (14.5)	7 (10.6)	1.6 0.6–4.1	0.4 0.3–5.4	1.8 0.5–6.7	0.3	0.6	0.3
A	63 (59.4)	27 (58.7)	36 (60.0)	152 (62.8)	47 (52.2)	85 (64.4)	1.00 (reference)	1.00 (reference)	1.00 (reference)			
G	43 (40.6)	19 (41.3)	24 (40.0)	90 (37.2)	43 (47.8)	47 (35.6)	1.1 0.7–1.8	0.8 0.4–1.6	1.2 0.7–2.2	0.6	0.5	0.6
	Vascular involvement											
	Positive			Negative			OR (95% CI)			*p*-value		
AA	5 (38.5)	3 (30.0)	2 (66.70	63 (38.7)	27 (39.1)	36 (38.3)	1.00 (reference)	1.00 (reference)	1.00 (reference)			
AG	5 (38.5)	4 (40.0)	1 (33.3)	78 (47.9)	32 (46.4)	46 (48.9)	0.8 0.2–2.9	1.1 0.2–5.5	0.4 0.03–4.5	0.7	0.9	0.4
GG	3 (23.1)	3 (30.0)	0 (0.0)	22 (13.5)	10 (14.5)	12 (12.8)	1.7 0.4–7.8	2.7 0.5–15.6	0.6 0.03–13.01	0.5	0.3	0.7
A	15 (57.7)	10 (50.0)	5 (83.3)	204 (62.6)	86 (62.3)	118 (62.8)	1.00 (reference)	1.00 (reference)	1.00 (reference)			
G	11 (42.3)	10 (50.0)	1 (16.7)	122 (37.4)	52 (37.7)	70 (37.2)	1.2 0.5–2.7	1.6 0.6–4.2	0.3 0.04–2.9	0.6	0.3	0.3
	Cardiac involvement											
	Positive			Negative			OR (95% CI)			*p*-value		
AA	3 (27.3)	1 (14.3)	2 (50.0)	63 (39.1)	34 (37.8)	29 (40.8)	1.00 (reference)	1.00 (reference)	1.00 (reference)			
AG	5 (45.5)	4 (57.1)	1 (25.0)	76 (47.2)	45 (50.0)	31 (43.7)	1.4 0.3-6.0	3.02 0.3–28.3	0.5 0.04–5.4	0.7	0.3	0.5
GG	3 (27.3)	2 (28.6)	1 (25.0)	22 (13.7)	11 (12.2)	11 (15.5)	0.9 0.5–15.2	6.2 0.5–74.9	1.3 0.1–16.0	0.2	0.1	0.8
A	11 (50.0)	6 (42.8)	5 (62.5)	202 (62.7)	113 (62.8)	89 (62.7)	1.00 (reference)	1.00 (reference)	1.00 (reference)			
G	11 (50.0)	8 (57.2)	3 (37.5)	120 (37.3)	67 (37.2)	53 (37.3)	1.7 0.7-4.0	2.2 0.7–6.8	1.0 0.2–4.4	0.2	0.15	0.9
	Lung involvement											
	Positive			Negative			OR (95% CI)			*p*-value		
AA	1 (16.7)	0 (0.0)	1 (33.3)	46 (36.5)	24 (36.9)	22 (36.1)	1.00 (reference)	1.00 (reference)	1.00 (reference)			
AG	4 (66.7)	3 (100)	1 (33.3)	61 (48.4)	30 (46.2)	31 (50.8)	3.01 0.3–27.9	5.6 0.3-111.1	0.7 0.04–11.9	0.3	0.3	0.8
GG	1 (16.7)	0 (0.0)	1 (33.3)	19 (15.1)	11 (16.9)	8 (13.1)	2.4 0.1–40.7	2.1 0.04–114.2	2.7 0.15–49.4	0.5	0.7	0.5
A	6 (50.0)	3 (50.0)	3 (50.0)	153 (60.7)	78 (60.0)	75 (61.5)	1.00 (reference)	1.00 (reference)	1.00 (reference)			
G	6 (50.0)	3 (50.0)	3 (50.0)	99 (39.3)	52 (40.0)	47 (38.5)	1.5 0.5–4.9	1.5 0.3–7.7	1.6 0.3–8.2	0.5	0.6	0.6
	Renal involvement											
	Positive			Negative			OR (95% CI)			*p*-value		
AA	8 (44.4)	3 (42.9)	5 (45.5)	59 (37.1)	25 (35.2)	34 (38.6)	1.00 (reference)	1.00 (reference)	1.00 (reference)			
AG	8 (44.4)	3 (42.9)	5 (45.5)	76 (47.8)	33 (46.5)	43 (48.9)	0.8 0.3–2.2	0.7 0.14–4.1	0.8 0.2–2.9	0.6	0.7	0.7
GG	2 (11.1)	1 (14.3)	1 (9.1)	24 (15.1)	13 (18.3)	11 (12.5)	0.6 0.1–3.1	0.6 0.1–6.8	0.6 0.1–5.9	0.6	0.7	0.7
A	24 (66.7)	9 (64.3)	15 (68.2)	194 (61.0)	83 (58.4)	111 (63.1)	1.00 (reference)	1.00 (reference)	1.00 (reference)			
G	12 (33.3)	5 (35.7)	7 (31.8)	124 (39.0)	59 (41.6)	65 (36.9)	0.8 0.4–1.6	0.8 0.2–2.4	0.8 0.3–2.06	0.5	0.7	0.6
	Gastrointestinal involvement											
	Positive			Negative			OR (95% CI)			*p*-value		
AA	7 (41.2)	4 (66.7)	3 (27.3)	57 (38.8)	24 (34.8)	33 (42.3)	1.00 (reference)	1.00 (reference)	1.00 (reference)			
AG	6 (35.3)	1 (16.7)	5 (45.5)	70 (47.6)	33 (47.8)	37 (47.4)	0.7 0.2–2.2	0.2 0.02–1.7	1.5 0.3–6.7	0.5	0.1	0.6
GG	4 (23.5)	1 (16.7)	3 (27.3)	20 (13.6)	12 (17.4)	8 (10.3)	1.6 0.4–6.1	0.5 0.05–4.9	4.1 0.7–24.4	0.5	0.6	0.1
A	20 (58.8)	9 (75.0)	11 (50.0)	184 (62.6)	81 (58.7)	103 (66.0)	1.00 (reference)	1.00 (reference)	1.00 (reference)			
G	14 (41.2)	3 (25.0)	11 (50.0)	110 (37.4)	57 (41.3)	53 (34.0)	1.2 0.6–2.4	0.5 0.1–1.8	1.9 0.8–4.8	0.7	0.3	0.1
***TLR9*rs352140**												
	Oral aphthous											
	Positive			Negative			OR (95% CI)			*p*-value		
TT	44 (24.9)	21 (26.9)	23 (23.2)	0 (0.0)	0 (0.0)	0 (0.0)	1.00 (reference)	1.00 (reference)	1.00 (reference)			
CT	84 (47.5)	36 (46.2)	48 (48.5)	2 (66.7)	1 (50.0)	1 (100)	0.4 0.02–8.1	0.6 0.02–14.5	0.7 0.03–17.5	0.5	0.7	0.8
CC	49 (27.7)	21 (26.9)	28 (28.3)	1 (33.3)	1 (50.0)	0 (0.0)	0.4 0.01–9.3	0.3 0.01–8.6	1.2 0.02–63.5	0.5	0.5	0.9
T	172 (48.6)	78 (50.0)	94 (47.5)	2 (33.3)	1 (25.0)	1 (50.0)	1.00 (reference)	1.00 (reference)	1.00 (reference)			
C	182 (51.4)	78 (50.0)	104 (52.5)	4 (66.7)	3 (75.0)	1 (50.0)	0.5 0.9–2.9	0.3 0.03–3.3	1.1 0.07–17.9	0.5	0.3	0.9
	Genital aphthous											
	Positive			Negative			OR (95% CI)			*p*-value		
TT	24 (22.9)	12 (27.3)	12 (19.7)	16 (23.5)	8 (25.0)	8 (22.2)	1.00 (reference)	1.00 (reference)	1.00 (reference)			
CT	53 (50.5)	22 (50.0)	31 (50.8)	31 (45.6)	13 (40.6)	18 (50.0)	1.1 0.5–2.5	1.12 0.4–3.5	1.15 0.4–3.3	0.7	0.8	0.8
CC	28 (26.7)	10 (22.7)	18 (29.5)	21 (30.9)	11 (34.4)	10 (27.8)	0.9 0.4–2.1	0.6 0.2–2.1	1.2 0.4–3.9	0.8	0.4	0.8
T	101 (48.1)	46 (52.3)	55 (45.1)	63 (46.3)	29 (45.3)	34 (47.2)	1.00 (reference)	1.00 (reference)	1.00 (reference)			
C	109 (51.9)	42 (47.7)	67 (54.9)	73 (53.7)	35 (54.7)	38 (52.8)	0.9 0.6–1.4	0.7 0.4–1.4	1.1 0.6–1.9	0.7	0.4	0.8
	Skin involvement											
	Positive			Negative			OR (95% CI)			*p*-value		
TT	25 (29.1)	14 (30.4)	11 (27.5)	18 (20.5)	7 (22.6)	11 (19.3)	1.00 (reference)	1.00 (reference)	1.00 (reference)			
CT	34 (39.5)	18 (39.1)	16 (40.0)	49 (55.7)	17 (54.8)	32 (56.1)	0.5 0.2–1.05	0.5 0.2–1.6	0.5 0.2–0.4	0.07	0.3	0.2
CC	27 (31.4)	14 (30.4)	13 (32.5)	21 (23.9)	7 (22.6)	14 (24.6)	0.9 0.4–2.1	1.0 0.3–3.06	0.9 0.3–2.9	0.8	1.0	0.9
T	84 (48.8)	46 (50.0)	38 (47.5)	85 (48.3)	31 (50.0)	54 (47.4)	1.00 (reference)	1.00 (reference)	1.00 (reference)			
C	88 (51.2)	46 (50.0)	42 (52.5)	91 (51.7)	31 (50.0)	60 (52.6)	0.9 0.6–1.5	1.0 0.5–1.9	0.9 0.6–1.8	0.9	1.0	0.9
	Eye involvement											
	Positive			Negative			OR (95% CI)			*p*-value		
TT	13 (19.4)	7 (22.6)	6 (16.7)	24 (26.7)	10 (28.6)	14 (25.5)	1.00 (reference)	1.00 (reference)	1.00 (reference)			
CT	31 (46.3)	15 (48.4)	16 (44.4)	44 (48.9)	15 (42.9)	29 (52.7)	1.3 0.6–2.9	1.4 0.4–4.7	1.3 0.4-4.0	0.5	0.6	0.7
CC	23 (34.3)	9 (29.0)	14 (38.9)	22 (24.4)	10 (28.6)	12 (21.8)	1.9 0.8–4.7	1.3 0.3–4.8	2.7 0.8–9.3	0.1	0.7	0.1
T	57 (42.5)	29 (46.8)	28 (38.9)	92 (51.1)	35 (50.0)	57 (51.8)	1.00 (reference)	1.00 (reference)	1.00 (reference)			
C	77 (57.5)	33 (53.2)	44 (61.1)	88 (48.9)	35 (50.0)	53 (48.2)	1.4 0.9–2.2	1.3 0.6–2.2	1.7 0.9–3.1	0.1	0.7	0.09
	Joint involvement											
	Positive			Negative			OR (95% CI)			*p*-value		
TT	24 (21.6)	12 (27.3)	12 (17.9)	19 (27.5)	8 (22.9)	11 (32.4)	1.00 (reference)	1.00 (reference)	1.00 (reference)			
CT	56 (50.5)	19 (43.2)	37 (55.2)	31 (44.9)	18 (51.4)	13 (38.2)	1.4 0.7–3.01	0.7 0.2–2.1	2.6 0.9–7.3	0.3	0.5	0.07
CC	31 (27.9)	13 (29.5)	18 (26.9)	19 (27.5)	9 (25.7)	10 (29.4)	1.3 0.6–2.9	0.9 0.3–3.3	1.6 0.5–5.1	0.5	0.9	0.4
T	104 (46.8)	43 (48.9)	61 (45.5)	69 (50.0)	34 (48.6)	35 (48.5)	1.00 (reference)	1.00 (reference)	1.00 (reference)			
C	118 (53.2)	45 (51.1)	73 (54.5)	69 (50.0)	36 (51.4)	33 (51.5)	1.1 0.7–1.7	0.9 0.5–1.8	1.3 0.7–2.3	0.6	0.9	0.4
	Neural involvement											
	Positive			Negative			OR (95% CI)			*p*-value		
TT	15 (28.3)	8 (34.8)	7 (23.3)	29 (24.0)	13 (23.6)	16 (24.2)	1.00 (reference)	1.00 (reference)	1.00 (reference)			
CT	24 (45.3)	9 (39.1)	15 (50.0)	60 (49.6)	27 (49.1)	33 (50.0)	0.8 0.3–1.7	0.54 0.2–1.7	1.04 0.3–3.05	0.5	0.3	0.9
CC	14 (26.4)	6 (26.1)	8 (26.7)	32 (26.4)	15 (27.3)	17 (25.8)	0.8 0.3-2.0	0.6 0.2–2.4	1.07 0.3–3.6	0.7	0.5	0.9
T	54 (50.9)	25 (54.3)	29 (48.3)	118 (48.8)	53 (48.1)	65 (49.2)	1.00 (reference)	1.00 (reference)	1.00 (reference)			
C	52 (49.1)	21 (45.7)	31 (51.7)	124 (51.2)	57 (51.9)	67 (50.8)	0.9 0.6–1.4	0.8 0.4–1.6	1.04 0.6–1.9	0.7	0.5	0.9
	Vascular involvement											
	Positive			Negative			OR (95% CI)			*p*-value		
TT	4 (30.8)	4 (40.0)	0 (0.0)	38 (23.3)	17 (24.6)	21 (22.3)	1.00 (reference)	1.00 (reference)	1.00 (reference)			
CT	3 (23.1)	2 (20.0)	1 (33.3)	83 (50.9)	35 (50.7)	48 (51.1)	0.3 0.07–1.6	0.2 0.04–1.4	1.3 0.05–33.9	0.2	0.12	0.9
CC	6 (46.2)	4 (40.0)	2 (66.7)	42 (25.8)	17 (24.6)	25 (26.6)	1.3 0.3–5.2	1.0 0.2–4.7	4.2 0.2–92.7	0.6	1.0	0.4
T	11 (42.3)	10 (50.0)	1 (16.7)	159 (48.8)	69 (50.0)	90 (47.9)	1.00 (reference)	1.00 (reference)	1.00 (reference)			
C	15 (57.7)	10 (50.0)	5 (83.7)	167 (51.2)	69 (50.0)	98 (52.1)	1.3 0.6–2.9	1.0 0.4–2.6	4.6 0.5-40.06	0.5	1.0	0.17
	Cardiac involvement											
	Positive			Negative			OR (95% CI)			*p*-value		
TT	3 (27.3)	2 (28.6)	1 (25.0)	39 (24.2)	19 (26.8)	20 (22.2)	1.00 (reference)	1.00 (reference)	1.00 (reference)			
CT	5 (45.5)	3 (42.9)	2 (50.0)	77 (47.8)	33 (46.5)	44 (48.9)	0.8 0.2–3.7	0.9 0.1–5.6	0.9 0.08–10.6	0.8	0.9	0.9
CC	3 (27.3)	2 (28.6)	1 (25.0)	45 (28.0)	19 (26.8)	26 (28.9)	0.9 0.2–4.5	1.0 0.1–7.8	0.8 0.04–13.1	0.9	1.0	0.8
T	11 (50.0)	7 (50.0)	4 (50.0)	155 (48.1)	71 (50.0)	84 (46.7)	1.00 (reference)	1.00 (reference)	1.00 (reference)			
C	11 (50.0)	7 (50.0)	4 (50.0)	167 (51.9)	71 (50.0)	96 (53.3)	0.9 0.4–2.2	1.0 0.3–2.9	0.9 0.2–3.6	0.9	1.0	0.8
	Lung involvement											
	Positive			Negative			OR (95% CI)			*p*-value		
TT	1 (16.7)	0 (0.0)	1 (33.3)	35 (27.8)	19 (29.2)	16 (26.20	1.00 (reference)	1.00 (reference)	1.00 (reference)			
CT	5 (83.3)	3 (100)	2 (66.7)	55 (43.7)	27 (41.5)	28 (45.9)	3.2 0.3–28.4	4.9 0.2-101.7	1.1 0.09–13.6	0.3	0.3	0.9
CC	0 (0.0)	0 (0.0)	0 (0.0)	36 (28.6)	19 (29.2)	17 (27.9)	0.3 0.01–8.2	1.0 0.02–52.9	0.3 0.01–8.3	0.5	1.0	0.5
T	7 (58.3)	3 (50.0)	4 (66.7)	125 (49.6)	65 (50.0)	60 (49.2)	1.00 (reference)	1.00 (reference)	1.00 (reference)			
C	5 (41.7)	3 (50.0)	2 (33.3)	127 (50.4)	65 (50.0)	62 (50.8)	0.7 0.2–2.3	1.0 0.2–5.1	0.5 0.08–2.7	0.5	1.0	0.4
	Renal involvement											
	Positive			Negative			OR (95% CI)			*p*-value		
TT	3 (16.7)	2 (28.6)	1 (9.1)	40 (25.2)	19 (26.8)	21 (23.9)	1.00 (reference)	1.00 (reference)	1.00 (reference)			
CT	9 (50.0)	4 (57.1)	5 (45.5)	76 (47.8)	32 (45.1)	44 (50.0)	1.6 0.4–6.2	1.2 0.2–7.1	2.4 0.3–21.7	0.5	0.8	0.4
CC	6 (33.3)	1 (14.3)	5 (45.5)	43 (27.0)	20 (28.2)	23 (26.1)	1.9 0.4–7.9	0.47 0.04–5.7	4.6 0.5–42.3	0.4	0.6	0.2
T	15 (41.7)	8 (57.1)	7 (31.8)	156 (49.0)	70 (49.3)	86 (48.9)	1.00 (reference)	1.00 (reference)	1.00 (reference)			
C	21 (58.3)	6 (42.9)	15 (68.2)	162 (51.0)	72 (50.7)	90 (51.1)	0.3 0.7–2.7	0.7 0.2–2.2	2.05 0.8–5.3	0.4	0.6	0.14
	Gastrointestinal involvement											
	Positive			Negative			OR (95% CI)			*p*-value		
TT	7 (41.2)	2 (33.3)	5 (45.5)	34 (23.1)	19 (27.5)	15 (19.2)	1.00 (reference)	1.00 (reference)	1.00 (reference)			
CT	6 (35.3)	2 (33.3)	4 (36.4)	73 (49.7)	32 (46.4)	41 (52.6)	0.4 0.12–1.3	0.6 0.08–4.6	3.4 0.8–14.4	0.1	0.6	0.11
CC	4 (23.5)	2 (33.3)	2 (18.2)	40 (27.2)	18 (26.1)	22 (28.2)	0.5 0.13–1.8	1.06 0.13–8.3	0.3 0.05–1.6	0.3	0.9	0.15
T	20 (58.8)	6 (50.0)	14 (63.6)	141 (47.9)	70 (50.7)	71 (45.5)	1.00 (reference)	1.00 (reference)	1.00 (reference)			
C	14 (41.2)	6 (50.0)	8 (36.4)	153 (52.1)	68 (49.3)	85 (54.5)	0.6 0.3–1.3	1.03 0.3–3.3	2.1 0.83–5.3	0.2	0.9	0.17

**Table 5:** *TLR9* gene haplotype distribution in Behcet patients and healthy controls

Haplotypes		Frequency		OR (95% CI)	*p*-value
rs187084	rs352140	Patients (2n = 410)	Controls (2n = 414)		
A	C	198 (48.4)	156 (37.7)	1.5 (1.2-2.0)	0.002
A	T	58 (14.1)	76 (18.3)	0.7 (0.5-1.0)	0.1
G	C	16 (3.9)	61 (14.7)	0.2 (0.1–0.4)	0.000
G	T	138 (33.6)	121 (29.3)	1.2 (0.9–1.6)	0.19

Data are presented as numbers (%)

*SNP* Single nucleotide polymorphism, *OR* Odds ratio, *CI* Confidence interval

## Discussion

Although the etiopathogenesis of BD is not completely understood, genetic predisposition, environmental factors, microbial agent triggers, endothelial cell dysfunction, neutrophil hyperfunction, and autoimmune mechanisms have been supposed [28, 29]. It is confirmed that the interaction between genetic and environmental factors plays a vital role in the pathogenesis of BD [30]. Genome-wide association studies (GWAS) support that single nucleotide gene polymorphisms might predispose individuals to such autoimmune diseases [31]. Recently some studies were published to point out the possible role of *TLR9* genetic variants rs352140 and rs187084, in the pathogenesis of some autoimmune diseases such as SLE, RA, AS, AML, and GD [17]- [24]. Here, in line with these studies, an association between rs352140 and rs187084 polymorphisms and BD was found among the Iranian population. To our knowledge, this is the first study to evaluate the possible role of *TLR9* in the pathogenesis of BD and its manifestations among Iranians. Our results revealed a significantly higher frequency of rs187084 AG and AG + GG genotypes in healthy subjects, as well as AG + GG genotype and G allele in healthy males. These findings indicate a protective role for these genotypes and alleles among Iranian individuals against BD. Nevertheless, a positive association between rs187087 AG genotype and G Allele and genital aphthous was found in male BD patients. Surprisingly, these results indicate that despite the protective role of the AG genotype against BD, it might increase the risk of genital aphthous in male BD patients. Besides, a significantly higher frequency of rs352140 CT and CT + TT genotypes was shown in healthy individuals, especially healthy females than in BD patients. Again, it is concluded that these genotypes and alleles protect Iranian individuals from BD. There have been limited investigations into the association between *TLR9* genotypes and the risk of BD. In a study by Ito et al., no significant association between rs187084 and rs352140 and susceptibility to BD has been found among Japanese individuals [12]. Fang et al. also evaluated these SNPs among Chinese BD patients and reached no significant association [32]. However, a study by Sakamoto et al. revealed a significantly higher frequency of rs352140 CC genotype among Japanese BD patients compared to the healthy control [33]. Furthermore, Dhifallah et al. showed an approximate to significant level difference for rs187084 TT genotype and T allele (*p* = 0.07 and *p* = 0.08, respectively) among Tunisian BD patients compared with the healthy subjects [34]. The differences in ethnicity, environmental factors that may impact gene expression, clinical heterogeneity, and limited sample size could explain the discrepancies between our findings and those of other studies. One limitation of the study is the absence of serum levels of TLR-9 that could improve the quality of the study.

## Conclusion

In summary, our study indicated that rs187084 AG and AG + GG genotypes and rs352140 CT and TT + CT genotypes protect Iranian individuals from BD but rs187084 AG genotype and G allele predispose male BD individuals to genital aphthous. However, additional studies are required to verify these results. Besides, further functional and linkage analyses are necessary to elucidate the precise role of rs187084 and rs352140 in the pathogenesis of BD.

### Supplementary Information

**Supplementary Material 1.**

## Data Availability

The datasets used and/or analyzed during the current study are available from the corresponding author upon reasonable request.
