# Supplementary material for: Toll-like receptor 9 (TLR9) genetic variants rs187084 and rs352140 confer protection from Behcet’s disease among Iranians
Source: BMC Rheumatol. 2024 Mar 14;8:13. doi: 10.1186/s41927-024-00382-x (PMC10938651; doi:10.1186/s41927-024-00382-x)

**Fig 1.** The genotypes of TLR9 single nucleotide polymorphisms. A) rs187084 and B) rs352140 genotypes. (L: Ladder, P: Patient).


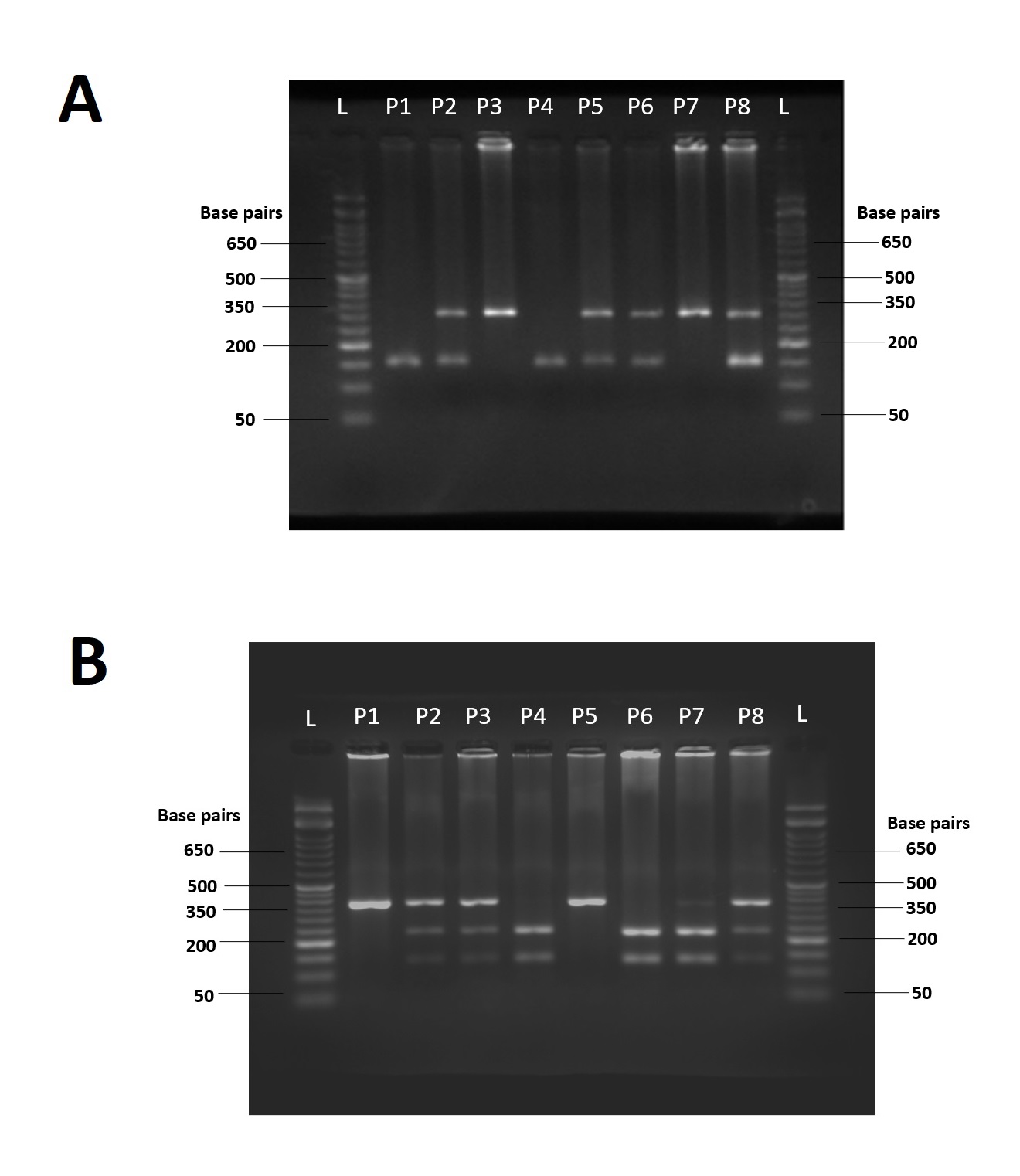


**Original gel images of rs 352140 and rs187084**


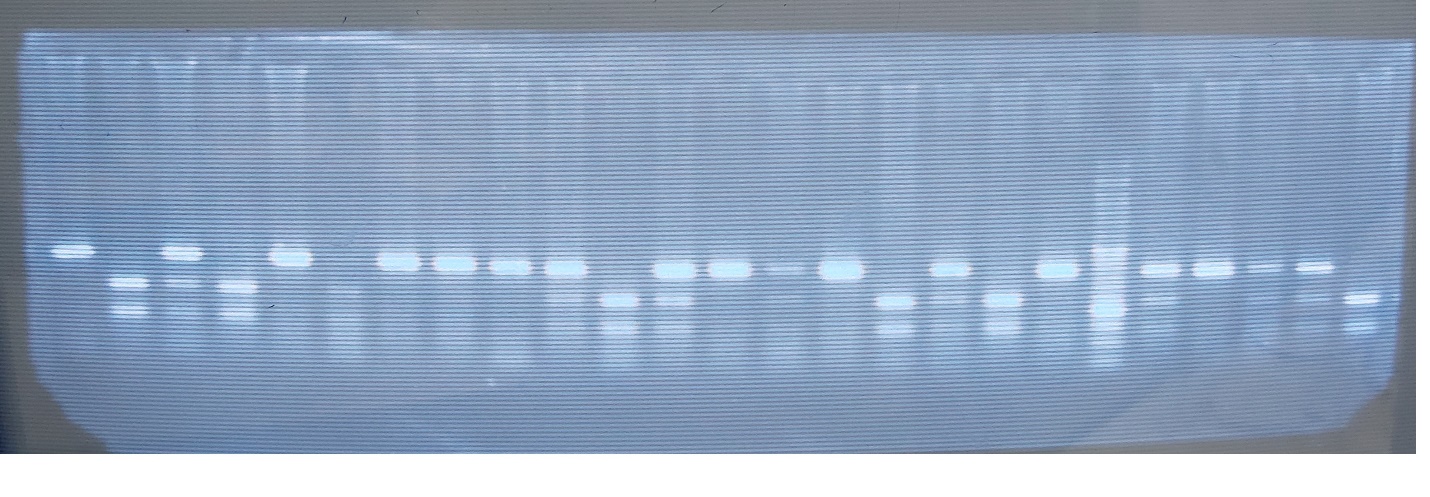

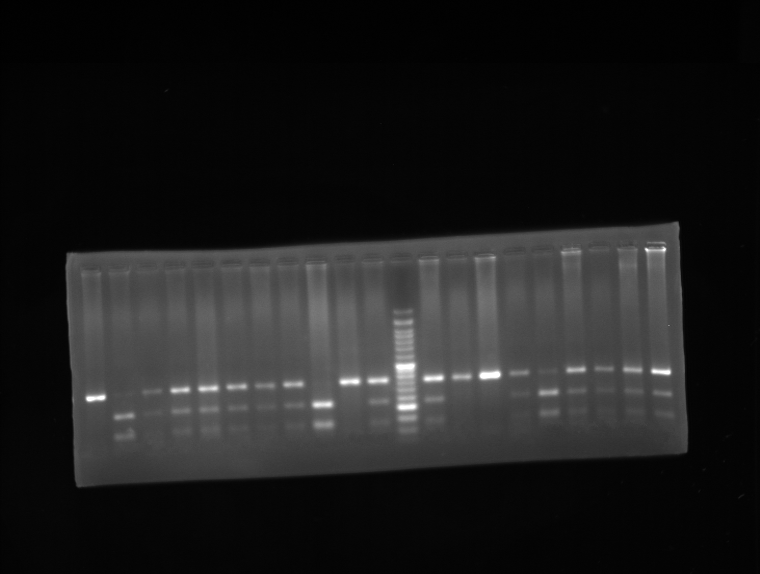

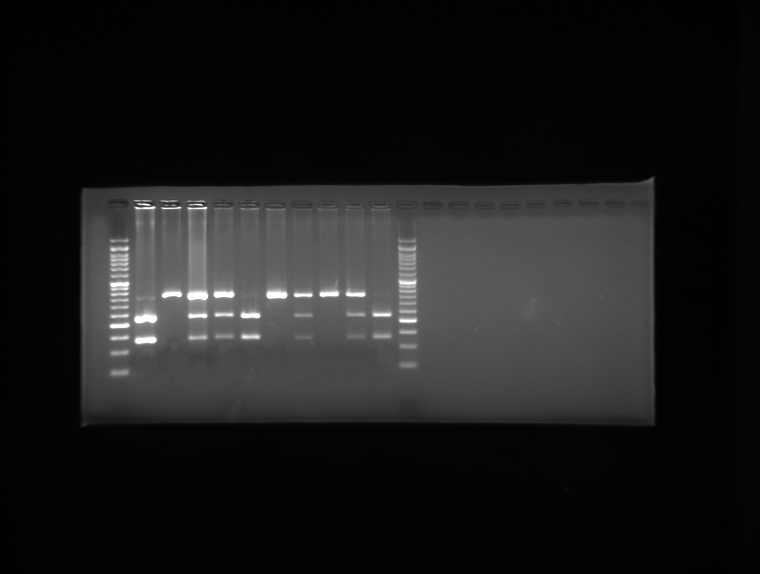

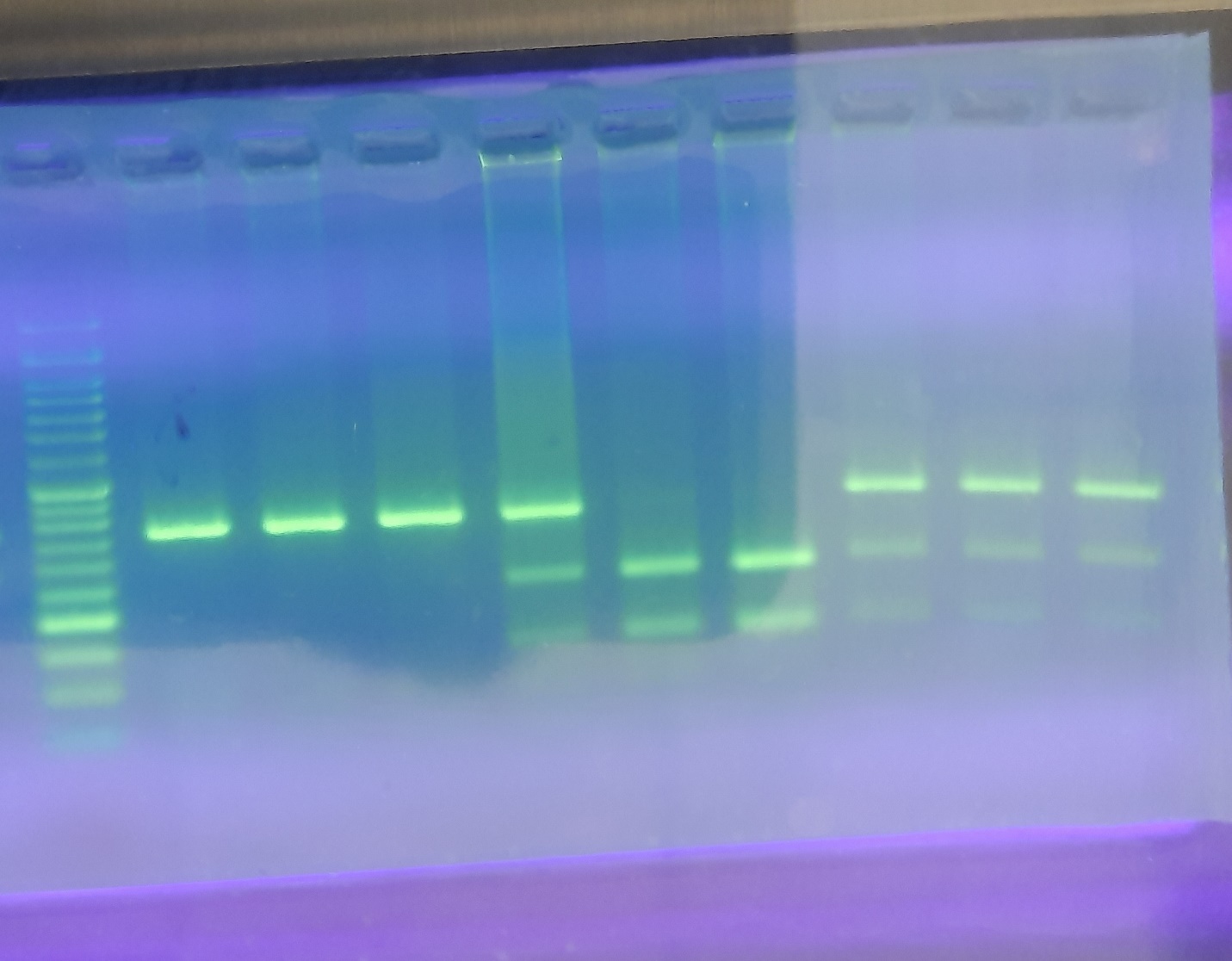

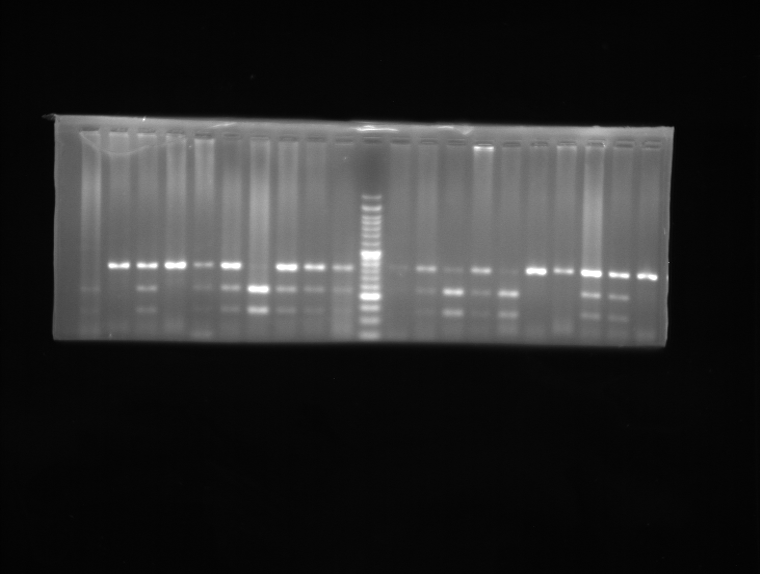

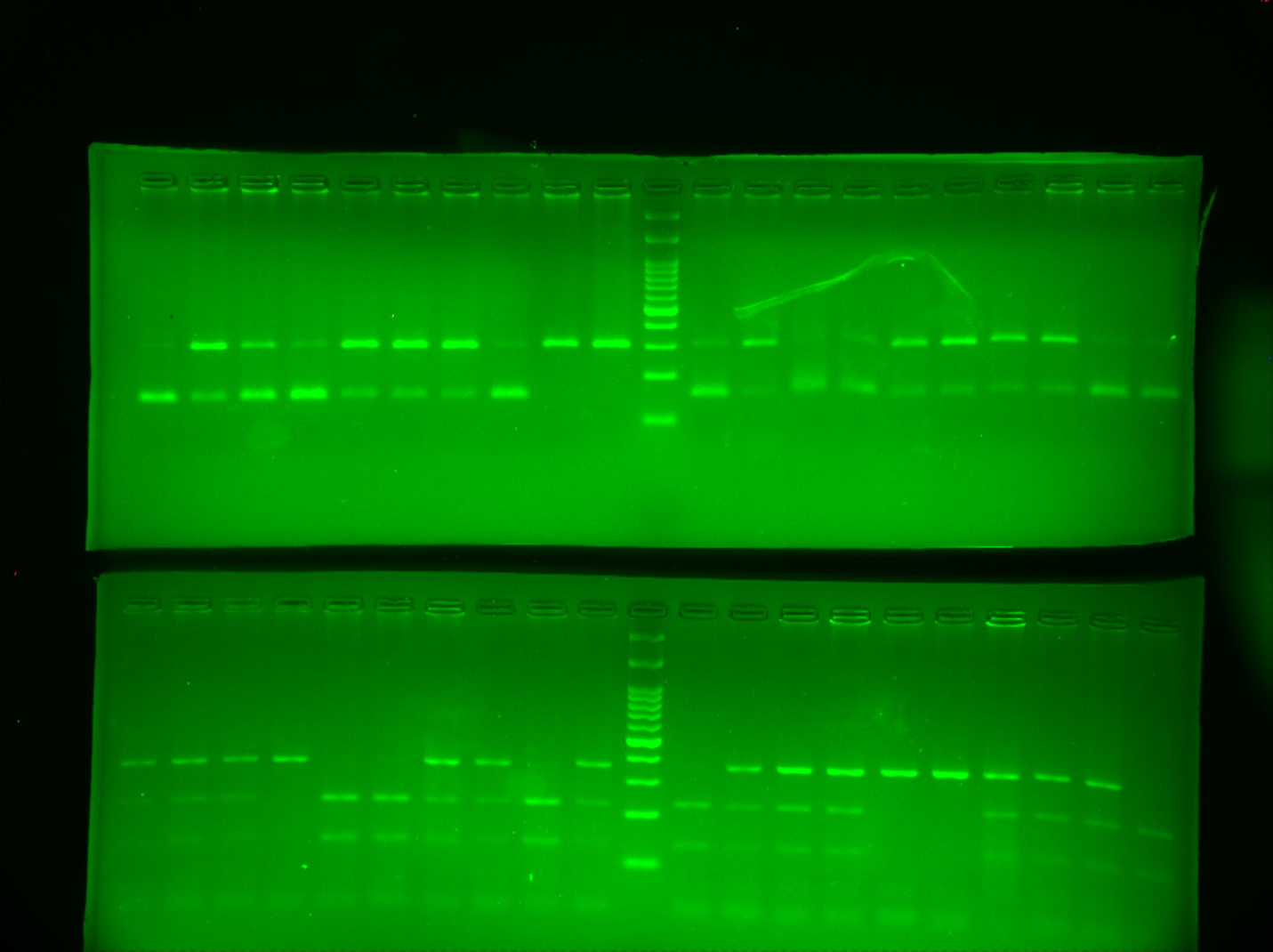

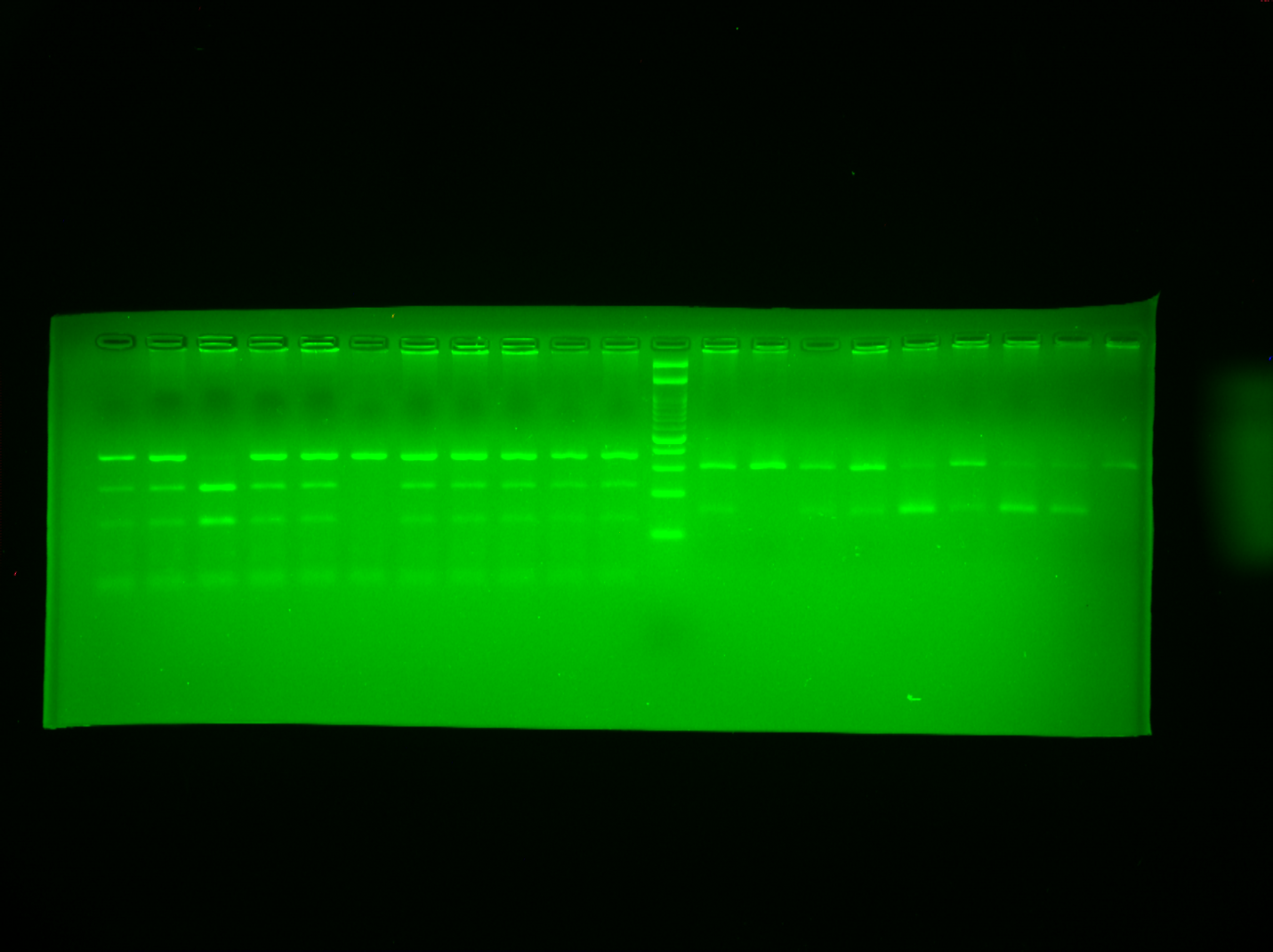

Supplement: Supplementary file 1 — Supplementary Material 1. [file 41927_2024_382_MOESM1_ESM.docx]
